# Attributing responsibility to farmers for environmental protection and climate action: insights from the European Union

**DOI:** 10.1007/s13412-024-00981-7

**Published:** 2024-09-30

**Authors:** Jale Tosun, Simon Schaub, Charlene Marek, Laura Kellermann, Marcus A. Koch

**Affiliations:** 1 Institute of Political Science and Heidelberg Center for the Environment, Heidelberg University, Bergheimer Straße 58, 69115 Heidelberg, Germany; 2 Department of Political Science, University of Oslo, Blindern, P.O. Box 1097, 0317 Oslo, Norway; 3 Institute of Political Science, Heidelberg University, Bergheimer Straße 58, 69115 Heidelberg, Germany; 4 Centre for Organismal Studies (COS), Heidelberg University, Im Neuenheimer Feld 345, 69120 Heidelberg, Germany; 5 Centre for Organismal Studies (COS) and Heidelberg Center for the Environment, Heidelberg University, Im Neuenheimer Feld 345, 69120 Heidelberg, Germany

**Keywords:** Climate change, Environmental degradation, European Union, Farmers, Responsibility

## Abstract

The attribution of responsibility is an important aspect of democratic government and governance. This study is interested in explaining variation in the responsibility that the public attributes to farmers for tackling climate change and environmental degradation. It analyzes data for respondents based in the 27 member states of the European Union as offered by a special issue of the Eurobarometer. Theoretically, the study postulates that if individuals perceive agriculture as a cause of climate change and environmental degradation, then they are more likely to indicate that it is a responsibility of farmers to take environmental and climate action. It also hypothesizes that individuals with a left-leaning ideology are more likely to attribute responsibility to farmers than right-leaning ones. Empirically, we find that the attribution of responsibility to farmers for mitigating climate change and environmental degradation is highest among Danes and lowest among Estonians. In 19 out of the 27 member states, our hypothesis holds true that individuals who perceive agriculture as a cause of climate change and environmental degradation are also more likely to attribute farmers’ responsibility for taking climate and environmental action. And in 11 member states, left-leaning individuals have a significantly higher likelihood of attributing farmers’ responsibility for tackling climate change and environmental degradation.

## Introduction

The controversy surrounding the Nature Restoration Law proposed by the European Commission as part of the measures for delivering on the European Green Deal (EGD) in 2023 and 2024 has exposed once more that some political actors see a divide between climate/environmental protection and agriculture. The Commission’s proposal stipulated restoration objectives for the recovery of nature in the land and sea areas of the European Union (EU) (European Commission 2022). The law not only concerns biodiversity but also aims to tackle climate change and introduce ecosystem services to prevent natural disasters and increase food security.

The Members of the European Parliament (MEPs) affiliated with the right-leaning European People’s Party (EPP) have been the most vocal critics of the proposal, stating that the “Nature Restoration Law has good intentions but would be a disaster for rural communities, farmers and fishermen and public authorities having to deal with the legal consequences” (European People’s Party Group 2023). Because of this opposition by the EPP-affiliated MEPs, which is the largest group in the European Parliament, the proposal failed in three parliamentary committees but survived the vote in the parliamentary plenary and was adopted in an amended version by a knife edge.

Subsequently, the proposal got stuck in the Council of the EU as it failed to gain the qualified majority votes necessary for adoption. Surprisingly, the Council then passed it in June 2024 when the competent minister from Austria, Leonore Gewessler (Green Party), voted in favor of it. Her positive vote on the proposal was a noteworthy move as she deviated from the abstain-vote agreed on within the coalition government led by the Austrian People’s Party.

The Nature Restoration Law is not the only instance when EPP-affiliated MEPs voted against climate and environmental measures targeting agriculture during the legislation period spanning 2019–2024. Another example is the Commission’s proposal for a Regulation on the Sustainable Use of Plant Protection Products, which 299 MEPs rejected while 207 supported it and 212 abstained from voting. Among the MEPs rejecting the proposal were many members of the EPP (European Parliament 2023). Similar to the EPP’s rejection of the Nature Restoration Law, a German member of the EPP group said that the defeat of the proposal in parliament shows “that pesticide reduction needs to be done with farmers and not against them” (Brzeziński 2023). Regardless of whether one agrees with the EPP’s position, it is important to note that the MEPs justify their position by referring to the interests of farmers. In other words, the EPP contends that it is responsive to the attitudes of farmers toward proposed policy measures targeting their activities.

European farmers themselves demonstrated in drastic form that they do not perceive policymakers to respond to their needs and interests by organizing a wave of protests across several EU member states in late 2023 and early 2024. The farmers’ protests focused on an array of longstanding grievances, including low food prices, trade in agricultural products with non-EU states (such as, most importantly, Ukraine), the subsidy system established by the Common Agricultural Policy, which favors large producers, and the EU’s climate and environmental policy proposed or adopted in the frame of the EGD (Cokelaere & Brzeziński 2024). In March 2024, the EU Commission reacted to the—often disruptive—farmers’ protests and proposed a package of support measures that aim, among other things, to temper the proposed climate and environmental measures (European Commission 2024).

The concessions show that the EU Commission responded to the demands of the protesting farmers. However, according to the democratic politics perspective on responsive government (Däubler et al. 2018; Hobolt & Klemmemsen 2005; Page & Shapiro 1983; Stecker & Tausendpfund 2016), elected representatives should respond to collective public attitudes toward political issues rather than to the opinion or interests of individual groups. At first sight, the concessions for farmers certainly appear to be an instance of the latter. Nevertheless, the policy actions of the EU Commission and the governments of the EU member states could be regarded as legitimate if the broader public supports the farmers’ demands. And this was indeed the case with the farmer protests: Farmers were able to count on public support in many EU member states, such as France (Euronews 2024), although they did lose support in certain countries, such as the Netherlands, when protests escalated (DutchNews 2024).

We contend that whether the public supports policy measures to mitigate climate change and environmental degradation in agriculture or supports the farmers’ demands to downscale such measures depends, among other things, on whether the public attributes responsibility to farmers for tackling environmental degradation and climate change. Individuals who regard farmers as actors who can effectively address environmental degradation and climate change might be critical of their demands to temper climate and environmental measures, whereas those who do not attribute them this responsibility may be neutral or supportive vis-à-vis their demands.

In this article, we do not examine public opinion on the European farmers’ protests of 2023 and 2024 or the EGD-related policy measures targeting farmers—these issues are too recent, and we do not have appropriate data on them. Instead, we take this opportunity to analyze an existing dataset and develop an improved understanding of European citizens’ attribution of responsibility to farmers for environmental degradation and climate change. This perspective is inspired by several works of literature, including the literature which has alluded to the importance of responsibility attribution and responsibility practices for climate and environmental action (Milford et al. 2022; Moos & Arndt 2023). Our analysis is based on the Special Eurobarometer 504, which was fielded in the second half of 2020 and represents a unique data source for learning about citizens’ attitudes toward farmers in the 27 EU member states.

We strive to answer three research questions. First, do individuals who attribute responsibility to farmers for causing environmental degradation and climate change also expect them to take action to protect the environment and mitigate global warming? Second, what individual-level factors explain the variation in this attribution of treatment responsibility across the EU member states? In this context, we are particularly interested in the degree to which ideological differences play a role. After all, the Commission’s proposals for implementing the EGD were challenged by a right-leaning political group, the EPP, and so it will be insightful to assess how important ideological differences among the citizenry are. Considering that the representation of farmers’ interests through parties differs among member states, our third research question is whether there exist differences in the attribution of responsibility to farmers among member states.

The remainder of this study unfolds as follows. In the next section, we provide background information on agriculture in the EU and the existence of Agrarian and Ecological parties in the EU member states. We then present our conceptual framework and derive hypotheses to explain citizen perceptions, which is followed by clarifications on the operationalization of the variables and the research design. Subsequently, we present our findings, discuss them, and provide some concluding remarks.

## Background information on the EU member states

**Fig. 1:**
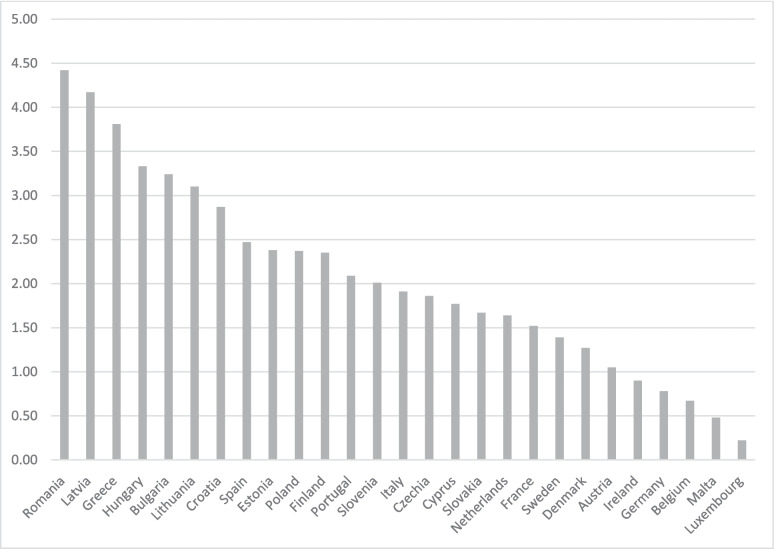
Value added in the agricultural sector as percentage of GDP, 2019. Notes: Data taken from: https://www.theglobaleconomy.com/rankings/share_of_agriculture/European-union/

The economic importance of agriculture varies starkly across the 27 EU member states. Figure 1 gives an overview of the value added in the agricultural sector as a percentage of the Gross Domestic Product (GDP) in 2019. The data reveal that Romania is the member state with the highest value (4.4% of the GDP) and Luxembourg with the lowest (0.2% of the GDP). More generally, the figure reveals that agriculture is an important source of income in the Eastern and Southern EU member states.

Of the member states in the Western and Northern parts of the EU, agriculture contributes considerably to the GDP of the Netherlands. There, political attempts to “green” agriculture have resulted in considerable turmoil and heavy protests by Dutch farmers in recent years (Leitheiser et al. 2022; van der Ploeg 2020). In fact, the political controversy around the greening of agriculture has resulted in the founding of a new political party in the Netherlands, the Farmer-Citizen Movement (*BoerBurgerBeweging;* BBB), which claims to represent the needs and interests of marginalized rural populations. In 2021, the founder of the BBB, Caroline van der Plas, won a seat at the general elections. In 2023, the BBB won the most seats in all provinces in the provincial elections and 16 seats in the Senate elections. In May 2024, the BBB announced that it would become a member of the multiparty coalition government led by the far-right Party for Freedom founded by Geert Wilders.

Party systems are widely considered to be stable since they date back to historically evolved societal cleavages (Lipset & Rokkan 1967). Against this backdrop, the emergence of a new political party can be considered as a sign that the existing political parties fail to address certain issues that citizens regard as important. The emergence of the BBB is a particularly interesting case, because the urban–rural divide is a cleavage out of which political parties have been emerging since the days of the Industrial Revolution. Thus, in the Dutch case, this cleavage recently became re-activated or strengthened to the degree that a new party was founded, which then enjoyed electoral success.

Whether the BBB will prevail in the long run is a different question—research on political parties and party systems has shown that newly founded parties are prone to disappear after a while. Nonetheless, it is noteworthy that issues related to agriculture and rural communities have become so politicized in the Netherlands (Feindt et al. 2021; Mamonova & Franquesa 2020; van der Ploeg 2020).

Many parties active in EU member states can be assigned to historically determined cleavages, as identified by Lipset and Rokkan (1967). In some but not all member states exist Agrarian parties that represent the interests of farmers and rural populations. In member states where no Agrarian parties exist, right-leaning parties tend to represent the interests of farmers (Tosun 2017). Compared to other party families, Agrarian parties are not as widespread in the EU, which actually gives their existence even more weight. It shows that rural communities in these countries, including farmers, perceive the need to rely on a dedicated party as an articulation of their interests.

Table 1 gives an overview of the Agrarian political parties that were represented in the member states’ parliaments in 2020, which is the same year that the data were collected for the Special Eurobarometer 504. In Finland, Latvia, Lithuania, Poland, and Sweden, the Agrarian parties also held mandates in the national parliaments. In Bulgaria, Croatia, the Czech Republic, Estonia, Slovakia, and Slovenia, Agrarian parties existed but without parliamentary representation. The Comparative Political Data Set (Armingeon et al. 2023), which we used to identify the parties, does not list the BBB as a party that existed in 2020. This is the only datapoint for which we deviate from Armingeon et al. (2023), as we chose to add the BBB to Table 1.

**Table 1:** Overview of Agrarian and Ecological parties, 2020

Country	Agrarian party	Agrarian party: parliament seats in %	Ecological party	Ecological party: parliament seats in %
Austria			• Green Alternative • Liste Peter Pilz	14.2
Belgium			• ECOLO (Francophone) • Green (Flemish)	14
Bulgaria	• Bulgarian National People’s Union—official • Bulgarian National People’s Union—United • Bulgarian National People’s Union—Nikola Petkov	0		
Croatia	• Croatian Peasant Party	0		
Cyprus			• Cyprus Green Party	3.6
Czech Republic	• Alliance of the Farmers and the Countryside	0	• Green Party	0
Denmark			• The Alternative	2.9
Estonia	• Farmers’ Union • Estonian Coalition Party • Estonian Country People’s Party	0	• Estonian Greens	0
Finland	• Centre Party	15.5	• Green League	10
France			• Greens • Generation Ecology • Other Ecologists	0
Germany			• Greens/Alliance 90	9.4
Greece			• Ecologists Greens	0
Hungary			• Politics Can be Different	4
Ireland			• Green Party	7.5
Italy			• Greens	0
Latvia	• Latvian Farmers’ Union • Union of Greens and Farmers	11		
Lithuania	• Lithuanian Peasant’s Party • Lithuanian Farmers and Greens Union	22.7	• Lithuanian Green Party	0
Luxembourg			• Green Alternative • Green Left • Green Party	15
Malta				
Netherlands	• Farmer-Citizen Movement*	0.0	• Green Left • Party of Animals • Volt Netherlands	12.6
Poland	• Polish Peasant Party • Peasant Alliance PL • Self-Defence of Polish Republic	6.5		
Portugal			• Greens • Party for Animals and Nature	1.7
Romania			• Ecological Movement from Romania	0
Slovakia	• Alliance of Farmers and the Countryside	0	• Party of Greens • Party of Greens in Slovakia	0
Slovenia	• Slovenian People’s Party	0	• Greens of Slovenia	0
Spain				
Sweden	• Agrarian Party, Centre Party	8.9	• Green Party	4.6

Remarks: Data taken from Armingeon et al. (2023); the share of seats is cumulated for countries in which multiple Agrarian or Ecological parties exist. *For the Netherlands, Armingeon et al. do not list the BBB. We added it to the list and therefore deviate from the dataset in this one instance

Cramer (2016) has compellingly argued that in the United States, rural voters distrust politicians who say that they respect their values and allocate them a fair share of the resources at their disposal. While the urban–rural divide is not as marked in Europe as in the United States, we still find it plausible to expect the existence of Agrarian parties not only to indicate the existence of such a divide, but also to result in rural populations being more dismissive of policy proposals which are directed at them. Consequently, we presume that in countries in which Agrarian parties have parliamentary representation, people living in rural areas are more supportive of farmers and their needs than are people living in urban ones.

The most common parties are Conservative and Social Democratic ones, but so-called new cleavage parties, such as Ecological parties, have become more widespread in recent years (Ford & Jennings 2020). Therefore, Table 1 also shows in which EU member states Ecological parties exist. Naturally, Ecological parties can be expected to support policies that seek to mitigate climate change and protect the environment. The existence of such parties suggests that these issues are salient, potentially making the public less likely to protect the interests of farmers over climate action and environmental protection. This should particularly hold true if no Agrarian parties exist that may have created a more positive image of farmers and rural communities.

Based on both the varying existence of Agrarian and Ecological parties in the EU member states, we contend that it is worth assessing how individuals perceive of the responsibilities of farmers for tackling environmental degradation and climate change by means of a country-comparative design. A rich corpus of literature shows that people’s attitudes are affected by the context in which they live (M. Kenny & Luca 2021; Stadelmann-Steffen & Eder 2021; Stoeckel 2013). Consequently, our analysis focuses on individual-level factors and runs country-by-country analyses to capture adequately the specific contexts. We bolster our findings by running multilevel regressions, which do not merely control for specific contexts but model them.

## Theoretical framework

In this study, we test two hypotheses and include a whole battery of individual-level factors as control variables in the models. The outcome variable is the attribution of responsibility to farmers for protecting the environment and tackling climate change. Responsibility has two facets: causal responsibility and treatment responsibility (Iyengar 1989). The first is about who has caused a problem; the second is about who can (potentially) alleviate a problem.

We argue that rather than representing two separate dimensions, a relationship exists between the different types of responsibility as attributed by individuals. More precisely, we postulate that the attribution of treatment responsibility to actors depends on the attribution of causal responsibility. In the case at hand, we hypothesize that individuals who regard agriculture as a source of environmental degradation and climate change are more likely to attribute treatment responsibility to farmers since it is farmers who carry out agriculture.

- H1: Persons who regard agriculture as a source of environmental degradation and climate change are *more likely* to indicate environmental protection and the tackling of climate change as a responsibility of farmers.

The second focal explanatory variable refers to citizens’ political orientation, that is, their self-placement on a left–right scale, and aligns with the discussion in the previous section about political parties in general and Agrarian and Ecological parties in particular. Extensive research has shown that left-leaning individuals hold more favorable attitudes toward climate action (Hammar & Jagers 2006; Harring & Sohlberg 2017; Jagers et al. 2021; Tobler et al. 2012). However, this finding does not translate into a hypothesis on the attribution of responsibility for climate action to farmers. To motivate such a hypothesis, it is important to go back to Agrarian and Ecological parties. It is fair to contend that these two parties hold opposing views on farmers: Agrarian parties have their strongholds in rural communities and tend to support farmers by demanding and implementing policies that protect their income (Batory & Sitter 2004). In marked contrast, Ecological parties have their strongholds in urban communities and call for more ambitious environmental and climate policies (Dolezal 2010).

However, as Table 1 has shown, Agrarian and Ecological parties do not exist in all EU member states, or they exist but are politically uninfluential. In such cases, right-leaning parties tend to protect the interests of farmers and rural communities (Hartung 2020; Schaub 2021). This argument is backed by the positions of the EPP, as illustrated in the introduction to this article. Right-leaning parties more generally support the tempering of regulatory requirements imposed on the economy. At the same time, Agrarian parties, even if they exist, correspond to right-leaning parties. In fact, in the Nordic countries, such as Finland, Agrarian parties can be considered as right-wing populist parties (Norocel 2016). It follows that right-leaning persons are less likely to indicate that farmers should take environmental and climate action. Instead, we expect them to assign farmers a privileged position in the national economy and to shield them from pressures to “green” their practices.

- H2: Right-leaning persons are *less likely* to indicate environmental protection and climate change as a responsibility of farmers.

While we do not formulate a specific hypothesis on this, we additionally expect the existence of Agrarian and Ecological parties to moderate the impact of ideology on the individuals’ attribution of responsibility to farmers. Agrarian parties could lower the impact of ideology by creating positive images of farmers that are shared throughout society. Conversely, the same move could strengthen the impact of ideology as people disagree with this portrayal of farmers, which aligns with the basic concept of affective polarization (Renström et al. 2023). Likewise, Ecological parties could lower the impact of ideology by garnering widespread support for climate and environmental measures. However, a general consensus on climate and environment action could also alienate some segments of the population, increasing the impact of ideology.

## Operationalization of the key variables

For the individual-level variables, we rely exclusively on the Special Eurobarometer 504. This contains information on the characteristics of participating individuals, which assists us in explaining response variations between citizens. It should be noted that the Commission tends to commission a Special Eurobarometer for “testing the waters” before or upon proposing policy reforms (Haverland et al. 2018). The background to the Special Eurobarometer 504 is the EGD in general and the “Farm to Fork Strategy” in particular, which is one of the components of the EGD (Wesseler 2022). Consequently, the questions asked in the survey provide nuanced insights into Europeans’ views on agriculture and farmers.

From this perspective, and in combination with the fact that it offers data on respondents residing in all 27 member states, it serves as an ideal database for carrying out a country-comparative assessment of the individual-level factors that shape the public’s attitude toward farmers. These features compensate for certain caveats pertaining to Eurobarometer data, such as the unclear formulation of questions (they tend to be too long and complicated), issues regarding the translation of the questions into the different European languages, and the use of self-reporting as a technique for data collection (Gatto & Panarello 2022).

The outcome variable of this study is binary and indicates whether respondents agreed (coded as 1) or disagreed (coded as 0) with the statement that farmers are responsible for protecting the environment and tackling climate change.

The first focal explanatory variable, *Cause*, gauges whether the respondents agreed with the statement that agriculture is one of the major causes of climate change. The second focal explanatory variable, *Ideology*, captures the ideological self-placement of individuals on a 10-point scale ranging from 1 (left) to 10 (right). The operationalization of this variable is established and widely used by other survey questionnaires as well, such as the European Social Survey.

The subsequent variables are all controls added to the models on the basis of pertinent studies that allude to their importance for explaining variation in outcome data. The data for the additional individual-level variables included in our analysis come from the same Eurobarometer dataset.

*Place of residence* indicates whether a respondent is based in a rural, peri-urban, or urban area. Empirical research has shown that the place of residence matters for individuals’ opinions on farmers (Howley et al. 2014; M. Kenny & Luca 2021; Tosun et al. 2024). *Age* captures all sorts of potentially intervening factors, such as support for climate action, professional status, and materialist vs. post-materialist values (Mostafa 2013). *Gender* is included to control for potential gender effects, which have been reported widely in the literature. Women tend to be more aware of sustainability issues (Mostafa 2013). Income is also a classic variable associated with attitudes toward sustainability (Lazaric et al. 2020). Here, we use an innovative measurement of income offered by the Eurobarometer data: whether respondents had difficulties paying their bills (*Difficulties paying bills*) in the last year (Tosun et al. 2024). Likewise, the dataset offers information on respondents’ self-assignment to a *Social class*. Another control variable refers to respondents’ *Education*, which has been identified as an important factor for explaining sustainability-related attitudes and behavior (Lazaric et al. 2020). The variable *Children* is binary and indicates whether children younger than 10 years live in a respondent’s household (Milfont et al. 2020).

Table 2 presents the descriptive statistics of the variables that entered the logistic regression models, which we estimated in order to account for the binary nature of the outcome variable. Table 2 also provides information on the share of missing information and on the response categories for the individual variables.

**Table 2:** Descriptive statistics

	Response categories									
Cause	*Agree*	*Disagree*								*Missing values % (n)*
	45.23	48.69								6.08 (1656)
Place of residence	*Rural area or village*	*Small/middle town*	*Large town*							
	33.66	36.06	30.24							0.04 (11)
Gender	*Male*	*Female*								
	46.01	53.99								0
Difficulties paying bills	*Most of the time*	*From time to time*	*(Almost) never*							
	7.11	23.26	68.90							0.73 (198)
Social class	*Working class*	*Lower middle class*	*Middle class*	*Upper middle class*	*Upper class*					
	23.83	15.30	49.72	8.71	0.94					1.51 (412)
Education	*No/pre-primary*	*Primary*	*Lower secondary*	*Upper secondary*	*Post-secondary non-tertiary*	*Short-cycle tertiary*	*Bachelor*	*Master*	*Doctor*	
	0.76	4.75	16.82	34.95	7.90	8.01	12.34	13.09	1.24	0.14 (39)
Children	*No*	*Yes*								
	83.34	16.66								0
	*Median*	*Mean*	*SD*	*Minimum*	*Maximum*					
Ideology	5	5.33	2.09	1	10					9.41 (2563)
Age	52	50.64	17.70	15	99					0.01 (4)
Agrarian parties (% seats)	0	2.39	5.70	0	22.7					0
Ecological parties (% seats)	0	3.69	5.27	0	15					0

*N*(dataset) = 27,237; *N*(analysis) = 23,157; total observations with missing values = 4080

Given the varying contexts in which the respondents in the different EU member states live, we chose to estimate separate models for them. We assigned the models to three different groups, differentiating between countries in which (i) Ecological but no Agrarian parties are represented in parliament, (ii) Agrarian parties are represented in government, and (iii) neither Agrarian nor Ecological parties are in parliament. In addition, we employed multilevel mixed-effect logistic regression models with fixed-effect estimates at the individual level and random effects at the country level. The multilevel models include covariates capturing the seat share of Agrarian and Ecological parties in parliament in the countries in which the respondents were based in 2020 as reported by Armingeon et al. (2023).

## Presentation and discussion of the empirical findings

**Fig. 2:**
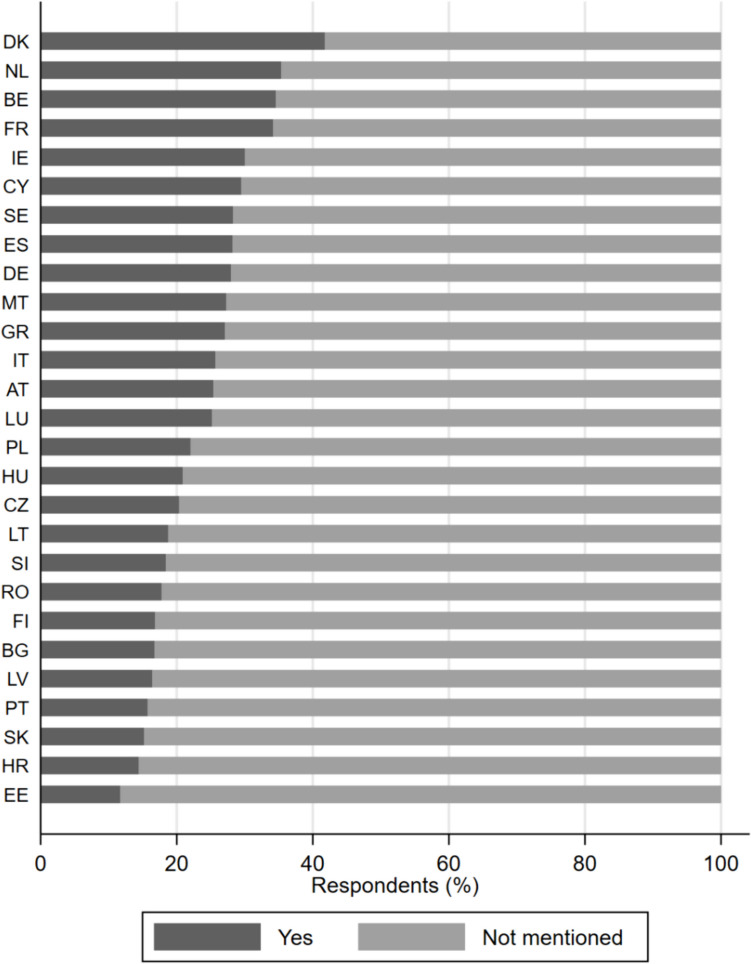
Country differences in how citizens perceive the treatment responsibility of farmers for environmental and climate protection

Before we turn to the results of our regression analysis, we need to look at our main outcome variable. Figure 2 shows that the share of citizens who attribute responsibility to farmers for taking action against environmental degradation and climate change differs substantially among EU member states. The share of respondents who agree that it is a main responsibility of farmers to protect the environment and tackle climate change is lowest in Estonia (12% agreement), which aligns with the more general observation that the largest share of countries where people do not perceive such action as a responsibility of farmers are the Central and Eastern European countries that joined the EU in 2004. Interestingly, Finnish respondents (17% agreement) also fall into this cluster, as the share of people connecting agricultural activities carried out by farmers to climate change and environmental degradation is low.

On the other end of the spectrum are the respondents from Denmark (42% agreement) and the Netherlands (35% agreement). The empirical picture for the Dutch respondents is particularly interesting given the recent emergence of the BBB. This hints at a polarization with regard to the attitudes that Dutch people hold on farmers and to what extent they should be targeted by regulatory measures to reduce their carbon and environmental footprint. Our observation aligns with research which has found that regional resentment exists in the Netherlands—it is stronger in rural areas than in urban ones (Lange et al. 2023).

Tables 3, 4, 5, 6 and 7 present the empirical results of the logistic regression models. The covariates associated with the outcome variable are presented in the form of odds ratios. Odds ratios greater than 1 indicate that an increase in a covariate’s value increases the odds of agreement with a given response option, whereas odds ratios smaller than 1 indicate a decrease in the odds.

**Table 3:** Countries with Ecological but no Agrarian parties in parliament (1)

Covariates	Austria	Belgium	Cyprus	Denmark	Germany	Hungary	Ireland
Cause	1.272	1.820^***^	1.579	2.123^***^	1.591^***^	1.259	1.974^***^
	(0.236)	(0.266)	(0.589)	(0.303)	(0.206)	(0.212)	(0.274)
Ideology	0.961	0.859^***^	0.935	0.865^***^	0.966	0.947	1.009
	(0.042)	(0.036)	(0.062)	(0.030)	(0.039)	(0.037)	(0.037)
Place of residence							
Rural area or village	*Reference category*						
Small/middle town	1.974^***^	0.675^**^	1.659	0.837	1.004	1.235	0.895
	(0.484)	(0.115)	(0.683)	(0.171)	(0.154)	(0.246)	(0.147)
Large town	1.376	0.766	4.859^***^	1.035	1.195	1.009	0.848
	(0.298)	(0.144)	(1.766)	(0.237)	(0.203)	(0.205)	(0.130)
Age	0.995	1.005	0.993	1.000	1.006	1.002	1.008^*^
	(0.006)	(0.004)	(0.010)	(0.005)	(0.004)	(0.005)	(0.005)
Gender							
Male	*Reference category*						
Female	0.910	1.024	0.608^*^	1.126	0.772^**^	1.207	0.859
	(0.164)	(0.147)	(0.179)	(0.161)	(0.100)	(0.201)	(0.111)
Difficulties paying bills							
Most of the time	*Reference category*						
From time to time	0.708	1.224	2.189	0.828	1.516	0.967	1.008
	(0.442)	(0.402)	(1.675)	(1.069)	(0.877)	(0.487)	(0.293)
Almost never/never	1.390	1.068	2.124	0.683	1.089	1.097	1.030
	(0.850)	(0.322)	(1.577)	(0.849)	(0.620)	(0.531)	(0.291)
Social class							
Working class	*Reference category*						
Lower middle class	1.231	0.938	4.068^**^	1.328	1.762^**^	1.402	1.300
	(0.456)	(0.274)	(2.366)	(0.433)	(0.399)	(0.364)	(0.283)
Middle class	0.953	1.123	2.194^*^	1.534^**^	1.557^**^	1.624^**^	1.280
	(0.297)	(0.276)	(0.879)	(0.334)	(0.313)	(0.399)	(0.235)
Upper middle class	0.948	1.381		1.573	1.477	1.523	1.020
	(0.404)	(0.450)		(0.436)	(0.413)	(0.854)	(0.272)
Higher class	0.962	4.082^*^		1.078	0.731	2.468	1.723
	(0.838)	(3.271)		(0.729)	(0.625)	(3.383)	(1.273)
Education							
No/pre-primary	3.273	0.677	0.504	0.522	2.154		0.870
	(4.546)	(0.530)	(0.464)	(0.337)	(2.998)		(1.003)
Primary		2.052^*^	1.002	1.895	1.156	0.830	1.858
		(0.882)	(0.540)	(1.548)	(0.605)	(0.673)	(1.000)
Lower secondary	1.286	0.757	0.800	0.732	0.858	0.716	1.769^*^
	(0.627)	(0.191)	(0.503)	(0.192)	(0.139)	(0.148)	(0.539)
Upper secondary	*Reference category*						
Post-secondary non-tertiary	1.371	0.419	0.709	0.605^**^	0.731	0.729	1.470
	(0.493)	(0.323)	(0.625)	(0.149)	(0.257)	(0.308)	(0.368)
Short-cycle tertiary	2.565^**^	1.295	0.881	1.029	0.903	0.333	1.316
	(0.968)	(0.277)	(0.454)	(0.216)	(0.298)	(0.255)	(0.321)
Bachelor/equivalent	3.771^**^	1.911^**^	0.710	0.807	1.359	0.966	1.235
	(2.158)	(0.570)	(0.339)	(0.237)	(0.327)	(0.259)	(0.260)
Master/equivalent	1.869	1.446^*^	0.644	1.036	1.490^**^	0.728	1.278
	(0.890)	(0.315)	(0.392)	(0.257)	(0.301)	(0.290)	(0.280)
Doctor/equivalent	3.956^**^	2.281		1.344	2.066^*^	2.659	1.675
	(2.703)	(1.399)		(0.763)	(0.839)	(2.903)	(0.738)
Children							
No children	*Reference category*						
Children	0.594^**^	0.707	0.854	0.851	0.885	0.914	0.986
	(0.156)	(0.167)	(0.349)	(0.187)	(0.177)	(0.211)	(0.162)
Observations	754	943	276	928	1347	959	1221

DV: farmer responsibility for environmental protection and climate change; exponentiated coefficients; standard errors in parentheses; *p < 0.10, **p < 0.05, ***p < 0.01

**Table 4:** Countries with Ecological but no Agrarian parties in parliament (2)

Covariates	Luxembourg	Netherlands	Portugal
Cause	1.323	2.642^***^	2.106^***^
	(0.265)	(0.378)	(0.547)
Ideology	0.822^***^	0.810^***^	0.917
	(0.051)	(0.033)	(0.060)
Place of residence			
Rural area or village	*Reference category*		
Small/middle town	1.518^*^	1.234	1.974^***^
	(0.339)	(0.196)	(0.484)
Large town	1.985^**^	1.157	1.647
	(0.546)	(0.224)	(0.510)
Age	1.006	1.009^*^	0.998
	(0.008)	(0.005)	(0.008)
Gender			
Male	*Reference category*		
Female	0.928	1.038	0.913
	(0.187)	(0.147)	(0.192)
Difficulties paying bills			
Most of the time	*Reference category*		
From time to time	0.690	0.895	1.058
	(0.330)	(0.468)	(0.380)
Almost never/never	0.698	0.855	1.396
	(0.320)	(0.343)	(0.500)
Social class			
Working class	*Reference category*		
Lower middle class	0.777	2.070^*^	0.804
	(0.346)	(0.883)	(0.258)
Middle class	0.789	2.029^**^	1.157
	(0.323)	(0.711)	(0.342)
Upper middle class	0.817	1.905^*^	2.069
	(0.381)	(0.710)	(1.489)
Higher class	0.618	2.704^**^	
	(0.468)	(1.309)	
Education			
No/pre-primary			0.255
			(0.268)
Primary	2.797	0.966	0.453^**^
	(1.770)	(0.389)	(0.159)
Lower secondary	1.323	1.368	0.570^*^
	(0.549)	(0.313)	(0.183)
Upper secondary	*Reference category*		
Post-secondary non-tertiary	1.319	1.025	4.699^**^
	(0.454)	(0.699)	(3.690)
Short-cycle tertiary	1.955^*^	1.129	0.435
	(0.770)	(0.331)	(0.477)
Bachelor/equivalent	1.982^**^	1.432	0.769
	(0.665)	(0.320)	(0.239)
Master/equivalent	1.516	1.057	0.921
	(0.459)	(0.222)	(0.508)
Doctor/equivalent	0.911	0.795	1.349
	(0.652)	(0.441)	(1.430)
Children			
No children	*Reference category*		
Children	0.916	1.062	1.136
	(0.235)	(0.275)	(0.345)
Observations	581	1025	751

DV: farmer responsibility for environmental protection and climate change; exponentiated coefficients; standard errors in parentheses; *p < 0.10, **p < 0.05, ***p < 0.01

**Table 5:** Countries with Agrarian parties in parliament

Covariates	Finland	Latvia	Lithuania	Poland	Sweden
Cause	2.993^***^	1.749^***^	1.212	1.351^*^	2.735^***^
	(0.531)	(0.352)	(0.248)	(0.229)	(0.453)
Ideology	0.788^***^	0.985	1.060	0.999	0.826^***^
	(0.037)	(0.058)	(0.054)	(0.037)	(0.030)
Place of residence					
Rural area or village	*Reference category*				
Small/middle town	0.934	0.809	0.979	1.389^*^	1.475^*^
	(0.216)	(0.197)	(0.253)	(0.275)	(0.321)
Large town	1.000	0.547^**^	1.014	1.322	1.681^**^
	(0.232)	(0.135)	(0.259)	(0.268)	(0.384)
Age	1.002	0.991	1.003	1.006	0.987^***^
	(0.007)	(0.007)	(0.006)	(0.005)	(0.005)
Gender					
Male	*Reference category*				
Female	0.871	1.114	1.150	1.131	0.889
	(0.156)	(0.243)	(0.245)	(0.194)	(0.141)
Difficulties paying bills					
Most of the time	*Reference category*				
From time to time	2.373	0.631	1.594	2.113	0.364
	(1.552)	(0.322)	(0.900)	(1.647)	(0.247)
Almost never/never	2.666	0.734	1.353	2.194	0.410
	(1.641)	(0.353)	(0.754)	(1.667)	(0.250)
Social class					
Working class	*Reference category*				
Lower middle class	0.523^*^	0.426^*^	2.133^**^	1.038	1.578
	(0.182)	(0.206)	(0.637)	(0.261)	(0.487)
Middle class	0.713	0.991	2.049^**^	1.443^*^	1.711^**^
	(0.192)	(0.244)	(0.573)	(0.308)	(0.431)
Upper middle class	0.862	2.702^**^	2.106	2.412^**^	1.899^**^
	(0.304)	(1.312)	(1.228)	(0.925)	(0.570)
Higher class	0.660	0.664		2.081	0.546
	(0.388)	(0.750)		(1.023)	(0.504)
Education					
No/pre-primary			2.332		1.582
			(2.807)		(2.014)
Primary	1.306		0.899	0.952	3.738
	(0.776)		(0.564)	(0.935)	(3.265)
Lower secondary	1.457	0.799	0.424^**^	0.862	1.085
	(0.549)	(0.299)	(0.183)	(0.281)	(0.364)
Upper secondary	*Reference category*				
Post-secondary non-tertiary	0.524	1.498	0.609	0.947	0.582^*^
	(0.323)	(0.492)	(0.232)	(0.432)	(0.172)
Short-cycle tertiary	1.441	1.965^*^			0.854
	(0.469)	(0.751)			(0.206)
Bachelor/equivalent	2.041^**^	1.377	0.978	0.529	1.131
	(0.644)	(0.447)	(0.246)	(0.258)	(0.268)
Master/equivalent	1.914^*^	1.264	0.827	1.148	0.686
	(0.653)	(0.424)	(0.325)	(0.296)	(0.176)
Doctor/equivalent	3.238^**^		1.489	0.742	1.169
	(1.650)		(1.918)	(0.543)	(0.627)
Children					
No children	*Reference category*				
Children	1.073	0.838	1.457	1.108	0.726
	(0.322)	(0.218)	(0.457)	(0.243)	(0.153)
Observations	1069	720	658	926	980

DV: farmer responsibility for environmental protection and climate change; exponentiated coefficients; standard errors in parentheses; *p < 0.10, **p < 0.05, ***p < 0.01

**Table 6:** Countries without Ecological or Agrarian parties in parliament (1)

Covariates	Bulgaria	Croatia	Czechia	Estonia	France	Greece
Cause	1.692^***^	1.833^***^	1.464^**^	3.383^***^	1.375^*^	1.431^**^
	(0.317)	(0.354)	(0.251)	(0.703)	(0.230)	(0.246)
Ideology	0.922^*^	0.943	0.972	0.884^**^	0.948	0.950
	(0.041)	(0.041)	(0.036)	(0.044)	(0.043)	(0.045)
Place of residence						
Rural area or village	*Reference category*					
Small/middle town	1.489	0.870	1.287	0.910	1.795^***^	1.023
	(0.405)	(0.210)	(0.270)	(0.221)	(0.347)	(0.258)
Large town	1.694^**^	0.739	1.102	0.638	1.614^*^	0.992
	(0.451)	(0.186)	(0.255)	(0.174)	(0.399)	(0.187)
Age	0.994	1.003	1.008	0.984^**^	0.994	0.994
	(0.006)	(0.007)	(0.006)	(0.007)	(0.005)	(0.006)
Gender						
Male	*Reference category*					
Female	0.947	0.977	1.009	1.240	1.127	0.989
	(0.174)	(0.190)	(0.173)	(0.254)	(0.184)	(0.158)
Difficulties paying bills						
Most of the time	*Reference category*					
From time to time	1.216	1.245	1.384	0.491	1.584	1.107
	(0.307)	(0.539)	(0.650)	(0.249)	(0.623)	(0.219)
Almost never/never	1.235	0.991	1.124	0.698	2.085^**^	1.287
	(0.340)	(0.429)	(0.506)	(0.319)	(0.758)	(0.348)
Social class						
Working class	*Reference category*					
Lower middle class	1.045	0.923	1.353	0.543^*^	0.509^**^	1.527^*^
	(0.304)	(0.307)	(0.407)	(0.173)	(0.136)	(0.379)
Middle class	0.724	0.967	1.419	0.523^**^	0.550^***^	1.460
	(0.178)	(0.254)	(0.387)	(0.147)	(0.123)	(0.339)
Upper middle class	0.827	1.328	1.603	0.752	0.762	5.478^**^
	(0.473)	(0.583)	(0.672)	(0.279)	(0.268)	(4.081)
Higher class		5.700^**^		0.610	0.282	2.995
		(4.531)		(0.813)	(0.379)	(4.281)
Education						
No/pre-primary			2.162			
			(1.536)			
Primary	2.618				0.976	0.922
	(2.459)				(0.375)	(0.312)
Lower secondary	0.947	1.130	0.953	2.127^*^	1.192	1.241
	(0.311)	(0.412)	(0.362)	(0.920)	(0.293)	(0.339)
Upper secondary	*Reference category*					
Post-secondary non-tertiary			1.029	0.503	1.704	1.227
			(0.627)	(0.210)	(1.146)	(0.390)
Short-cycle tertiary		1.489		0.623	1.870^**^	
		(0.509)		(0.384)	(0.524)	
Bachelor/equivalent	0.800	1.008	1.249	0.947	1.553	1.001
	(0.262)	(0.634)	(0.452)	(0.263)	(0.496)	(0.208)
Master/equivalent	0.811	1.474	0.748	0.750	1.034	1.153
	(0.231)	(0.452)	(0.257)	(0.207)	(0.323)	(0.521)
Doctor/equivalent		1.934		0.333	1.855	1.491
		(1.626)		(0.337)	(0.911)	(1.537)
Children						
No children	*Reference category*					
Children	1.596^**^	2.103^***^	0.915	0.896	0.728	1.287
	(0.374)	(0.494)	(0.195)	(0.230)	(0.175)	(0.293)
Observations	845	944	878	1091	739	827

DV: farmer responsibility for environmental protection and climate change; exponentiated coefficients; standard errors in parentheses; *p < 0.10, **p < 0.05, ***p < 0.01

**Table 7:** Countries without Ecological or Agrarian parties in parliament (2)

Covariates	Italy	Malta	Romania	Slovakia	Slovenia	Spain
Cause	0.753^*^	2.466^***^	1.210	1.190	1.692^***^	1.857^***^
	(0.122)	(0.812)	(0.226)	(0.227)	(0.340)	(0.282)
Ideology	0.991	0.853^*^	0.930^*^	0.988	0.825^***^	0.872^***^
	(0.041)	(0.070)	(0.035)	(0.049)	(0.043)	(0.031)
Place of residence						
Rural area or village	*Reference category*					
Small/middle town	1.290	0.431^*^	0.878	1.213	0.882	0.987
	(0.429)	(0.189)	(0.197)	(0.260)	(0.239)	(0.180)
Large town	1.333	2.718^***^	1.035	1.371	1.281	0.703^*^
	(0.475)	(1.011)	(0.223)	(0.345)	(0.284)	(0.147)
Age	1.004	1.000	0.987^**^	0.990^*^	0.995	1.001
	(0.005)	(0.013)	(0.006)	(0.006)	(0.005)	(0.006)
Gender						
Male	*Reference category*					
Female	1.111	0.562^*^	0.997	1.342	1.658^**^	1.132
	(0.178)	(0.168)	(0.183)	(0.259)	(0.330)	(0.169)
Difficulties paying bills						
Most of the time	*Reference category*					
From time to time	2.186^*^	0.230^*^	0.979	0.550	1.527	0.739
	(0.890)	(0.174)	(0.372)	(0.339)	(0.722)	(0.207)
Almost never/never	2.595^**^	0.176^**^	0.751	0.647	1.607	0.678
	(1.058)	(0.134)	(0.288)	(0.383)	(0.671)	(0.172)
Social class						
Working class	*Reference category*					
Lower middle class	0.948	1.207	1.566	1.180	0.603	1.437
	(0.350)	(0.750)	(0.544)	(0.347)	(0.221)	(0.328)
Middle class	0.895	1.599	1.779^**^	0.991	0.690	0.922
	(0.315)	(0.836)	(0.446)	(0.258)	(0.158)	(0.172)
Upper middle class	0.809	2.001	1.258	1.108	0.896	1.206
	(0.352)	(2.387)	(0.531)	(0.672)	(0.417)	(0.403)
Higher class	0.962		1.321			
	(1.246)		(1.229)			
Education						
No/pre-primary						0.136^*^
						(0.152)
Primary	0.636	0.789	1.548	1.323		1.495
	(0.277)	(0.398)	(0.809)	(0.531)		(0.626)
Lower secondary	0.718	0.734	0.591^*^	0.984	0.929	0.839
	(0.162)	(0.348)	(0.175)	(0.244)	(0.232)	(0.340)
Upper secondary	*Reference category*					
Post-secondary non-tertiary	0.628	2.942^**^	2.092^*^		0.751	1.106
	(0.506)	(1.562)	(0.826)		(0.349)	(0.349)
Short-cycle tertiary	1.174	1.775	3.403		0.915	0.977
	(0.895)	(1.456)	(2.732)		(0.274)	(0.330)
Bachelor/equivalent	1.125	0.561	0.989	0.357	1.211	1.084
	(0.435)	(0.406)	(0.270)	(0.378)	(0.447)	(0.360)
Master/equivalent	1.052	1.164	4.235^***^	1.668^**^	0.566	1.267
	(0.259)	(0.985)	(1.799)	(0.431)	(0.266)	(0.454)
Doctor/equivalent					1.088	1.487
					(0.918)	(0.754)
Children						
No children	*Reference category*					
Children	0.889	1.049	1.054	0.678	1.054	0.891
	(0.244)	(0.522)	(0.234)	(0.185)	(0.309)	(0.174)
Observations	835	280	928	842	745	964

DV: farmer responsibility for environmental protection and climate change; exponentiated coefficients; standard errors in parentheses; *p < 0.10, **p < 0.05, ***p < 0.01

The regression tables group the countries according to the presence of Ecological and/or Agrarian parties in 2020. Tables 3 and 4 present the findings of the logistic regression models for countries in which an Ecological party was represented in parliament, but not an Agrarian party. Table 5 presents the findings for respondents based in countries where an Agrarian party was represented in parliament. This group comprises both countries in which only an Agrarian party was represented (e.g., Poland) as well as countries in which Agrarian parties were represented along with Ecological parties (e.g., Finland). Tables 6 and 7 then present the models estimated using data for respondents from countries in which neither an Agrarian nor an Ecological party held seats in parliament.

**Fig. 3:**
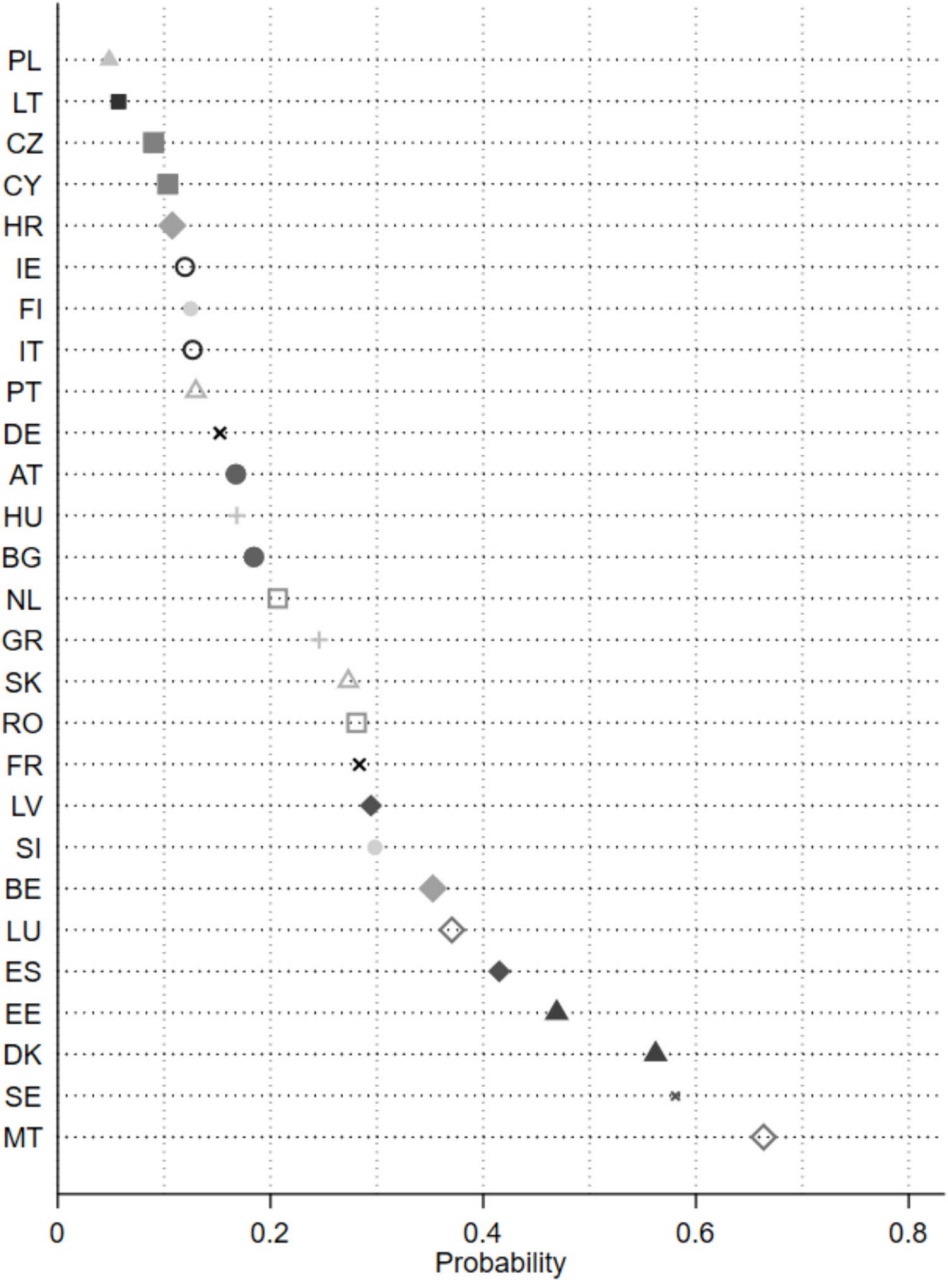
Probability to associate farmers with responsibility for climate change and environmental degradation for reference group. Note: Logistic regression intercept values (all predictor variables equal to zero or reference level), expressed as probabilities (*e*^β^_0_ / (1 + *e*^β^_0_))

Before turning to the findings of the regression models, Fig. 3 presents the intercepts for each of the 27 country-specific models. The intercept values as presented here report probabilities for a specific group of respondents with identical individual characteristics (reference group of the models) to indicate that farmers are responsible for tackling climate change and environmental degradation. We can infer from the figure that there is considerable variation across the respondents in the individual member states. The probability of attributing farmers this responsibility is lowest in Poland and highest in Malta for this specific group of respondents. The cross-country variation supports our choice to fit country-specific models.

In our first hypothesis, we postulated that causal and treatment responsibility are positively associated when asking individuals how they attribute responsibility to farmers for environmental degradation and climate change. Our empirical findings largely support this hypothesis. In 19 EU member states, individuals have significantly higher odds of perceiving environmental protection and climate action as a responsibility of farmers if they regard agriculture as a main cause of environmental degradation and global warming.

In countries with Ecological parties (Tables 3 and 4), only in Cyprus, Hungary, and Luxembourg are cause and treatment responsibility not statistically significantly associated. In the five countries with Agrarian parties represented in parliament (Table 5), the covariate is insignificant in Lithuania only. In the 12 countries with neither an Ecological or Agrarian party represented in parliament, the odds ratios for *Cause* are only insignificant in Romania and Slovakia.

Overall, the logistic regression models run separately for each country provide ample evidence for H1: they show a positive and statistically significant relationship between cause and treatment responsibility in 19 countries. Italy represents a puzzling case, as the odds ratio for the covariate is significant at the 10% level yet smaller than one. This means that there are individuals in Italy who have a lower likelihood of assigning farmers the responsibility of taking appropriate action, even though they agree that agriculture is a main cause of environmental degradation and climate change. This finding is counter-intuitive and makes it an interesting case for further research.

Turning to our second hypothesis, we find support for 11 member states that right-leaning individuals tend to have smaller odds of perceiving farmers as responsible for protecting the environment and mitigating climate change. Compared to the findings discussed previously, the number of member states with insignificant associations is larger. The odds ratios are significant for respondents based in Belgium, Denmark, Luxembourg, and the Netherlands in the first country cluster (Tables 3 and 4). In the second cluster, the odds ratios are only significant for Finland and Sweden. In the third cluster, the odds ratios are significant for respondents based in Bulgaria, Estonia, Malta, Romania, Slovenia, and Spain. It is worth remarking that in this cluster, the odds ratios are significant at the 10% level only in three models. In each country cluster, about 40% of the models support H2, suggesting that the findings vary across the clusters as much as they vary within them.

**Fig. 4:**
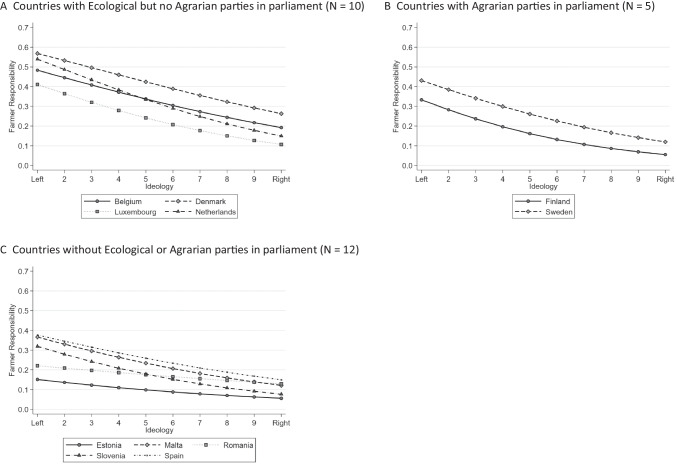
Predicted probabilities for the effect of ideology. **A**. Countries with Ecological but no Agrarian parties in parliament (N = 10), **B**. Countries with Agrarian parties in parliament (N = 5), **C**. Countries without Ecological or Agrarian parties in parliament (N = 12)

However, the dominance of cross-country variation over cross-cluster variation does not reject the importance of Agrarian and Ecological parties for explaining the attitudes of individuals. Figure 4 presents predicted probabilities for the three country clusters separately to show how much the likelihood of assigning farmers with responsibility for climate change and environmental degradation differs between the left end of the ideological spectrum and the right end. It includes only those countries for which the odds ratios of *Ideology* were statistically significant. Differences in the likelihood of attributing farmers’ responsibility are greatest among respondents in countries with Ecological but no Agrarian parties (panel A) represented in parliament, as indicated by the comparatively larger slopes of the curves between left- and right-leaning respondents. Especially left-leaning people show comparatively higher levels of likelihood of associating farmers with climate change and environmental degradation, in marked contrast to those on the very right.

In countries with Agrarian parties in government (panel B), the likelihood also differs noticeably between the left and the right ends of the ideological axis. Interestingly, the likelihood for right-leaning persons in these countries, who are more inclined to vote for Agrarian parties, tends to be lower. This holds true especially for Finland, where strongly right-leaning people have a likelihood of only 6% to perceive farmers as responsible. Left-leaning people also tend to be relatively less likely to attribute responsibility to farmers in these two countries (Finland and Sweden). The likelihood for a left-leaning person in Sweden is well below 40% and in Finland even below 30%.

In marked contrast, differences in the likelihood to attribute farmers’ responsibility are smaller between left- and right-leaning respondents based in countries in the third cluster (panel C), as indicated by the flatter slopes of the curves. In Estonia especially the difference between strongly left-oriented individuals and strongly right-oriented people is particularly small.

Collectively, the insights offered by Fig. 4 suggest that there are some cluster-specific effects of the ideology variable in terms of the strength of the effects, but not in respect of whether such effects exist.

Turning again to Tables 3, 4, 5, 6 and 7, the control variables also offer some interesting insights. *Place of residence* seems to matter in some EU member states; in most cases, it is individuals living in more urban areas that have a higher likelihood to perceive farmers as responsible for taking action. These countries include Austria, Bulgaria, Cyprus, France, Luxembourg, Malta, Poland, Portugal, and Sweden. This finding concurs with previous empirical findings reported by Tosun et al. (2024) on attitudes toward agriculture. It also aligns with more general attitudes—such as regional resentment, as reported by Lange et al. (2023)—and with social and political attitudes (M. Kenny & Luca 2021). Notable exceptions where these individuals seem to have a lower likelihood compared to people living in rural areas are Belgium, Latvia, and Spain.

*Age* has surprisingly low explanatory power, and in only a few countries is its association with the outcome variable statistically significant. In most of these cases, higher age reduces the likelihood of perceiving farmers as responsible—a finding we find plausible, especially considering the prominence of youth-based climate action movements such as Fridays for Future (Fritz et al. 2023).

Similarly, it is only in some countries that *Gender* is associated with perceptions of the responsibility in question. Contrary to our expectations, women seem to be less likely to attribute responsibility to farmers for taking action (except in Slovenia). Income (*Difficulties paying bills*) seems to be of minor importance for peoples’ views on farmers, as the associations are non-significant for most countries (only in France and Italy is lower income significantly associated with a lower likelihood to attribute farmers’ responsibility). However, self-assignment to a *Social class* appears to matter in most member states. Here, individuals who place themselves in higher social classes tend to have a higher likelihood to agree that farmers are responsible for taking action, which aligns with the gist of the concept of post-materialism (Mostafa 2013). Notable exceptions are France and Estonia, where people in higher social classes tend to have a lower likelihood. Similarly, individuals with higher *Education* tend to show higher agreement in some member states.

Finally, having *Children* appears to be of little importance for people’s views on whether farmers bear responsibility for environmental protection and climate mitigation. Exceptions are Bulgaria and Croatia, where people with children have a higher likelihood to agree that farmers have this responsibility, and Austria, where these people are less likely to view farmers as responsible.

In a last step, we present the findings of multilevel logistic regression models to test whether the existence of Agrarian and Ecological parties has a significant impact on the respondents’ views on farmers and their responsibility for tackling climate change and environmental degradation.

Table 8 presents three models: The first one includes a covariate capturing the existence of Agrarian parties only; the second model includes Ecological parties only; and the third includes covariates capturing both parties. The findings indicate that the representation of an Ecological party in parliament increases the odds for respondents to assign responsibility to farmers. The odds ratio is significant at the 10% level. We can observe the same for the third model, where the covariate for Ecological parties remains significant and greater than 1. The covariate for the representation of Agrarian parties in parliament is smaller than 1, but statistically insignificant in models 1 and 3. Nevertheless, the covariate shows a negative relationship, suggesting that in countries where Agrarian parties are present, respondents tend to perceive farmers as less responsible for taking climate and environmental action.

**Table 8:** Multilevel regression analysis

	Agrarian parties		Ecological parties		Both parties	
	Odds ratios	S.E	Odds ratios	S.E	Odds ratios	S.E
Cause	1.808^***^	0.156	1.805^***^	0.156	1.806^***^	0.156
Ideology	0.924^***^	0.011	0.924^***^	0.011	0.924^***^	0.011
Cause # ideology	0.990	0.015	0.990	0.015	0.990	0.015
Place of residence						
Rural area or village	*Reference category*					
Small/middle town	1.105^**^	0.044	1.106^**^	0.044	1.106^**^	0.044
Large town	1.128^***^	0.047	1.129^***^	0.047	1.129^***^	0.047
Age	0.998	0.001	0.998	0.001	0.998	0.001
Gender						
Male	*Reference category*					
Female	1.024	0.033	1.023	0.033	1.024	0.033
Difficulties paying bills						
Most of the time	*Reference category*					
From time to time	1.074	0.080	1.074	0.080	1.074	0.080
Almost never/never	1.118	0.081	1.115	0.081	1.116	0.081
Social class						
Working class	*Reference category*					
Lower middle class	1.130^**^	0.062	1.130^**^	0.062	1.129^**^	0.062
Middle class	1.145^***^	0.052	1.145^***^	0.052	1.144^***^	0.052
Upper middle class	1.264^***^	0.086	1.261^***^	0.086	1.260^***^	0.086
Higher class	1.304^*^	0.204	1.299^*^	0.203	1.299^*^	0.203
Education						
No/pre-primary	0.640^*^	0.158	0.641^*^	0.158	0.640^*^	0.158
Primary	0.944	0.088	0.947	0.088	0.946	0.088
Lower secondary	0.903^**^	0.047	0.903^**^	0.047	0.902^**^	0.047
Upper secondary	*Reference category*					
Post-secondary non-tertiary	0.952	0.065	0.947	0.065	0.948	0.065
Short-cycle tertiary	1.142^**^	0.072	1.136^**^	0.072	1.137^**^	0.072
Bachelor/equivalent	1.149^**^	0.062	1.146^**^	0.062	1.148^**^	0.062
Master/equivalent	1.108^*^	0.059	1.105^*^	0.059	1.106^*^	0.059
Doctor/equivalent	1.325^**^	0.173	1.322^**^	0.173	1.323^**^	0.173
Children						
No children	*Reference category*					
Children	0.953	0.043	0.953	0.043	0.953	0.043
Agrarian party seats	0.984	0.013			0.985	0.012
Ecological party seats			1.025^*^	0.014	1.024^*^	0.014
Variance	1.141^***^	0.043	1.132^***^	0.041	1.124^***^	0.038
*N* (member states)	27		27		27	
*N* (individuals	23,157		23,157		23,157	

DV: farmer responsibility for environmental protection and climate change; *p < 0.10, **p < 0.05, ***p < 0.01

The findings of the multilevel regression models provide further support for our hypotheses. The odds ratios for *Cause* and *Ideology* are significant in all three models and with the postulated sign. We also tested for an interaction effect between *Cause* and *Ideology*, but it turned out to be statistically insignificant.

To summarize, the presence of political parties matters for citizens’ attitudes on the responsibility they attribute to farmers for tackling climate change and environmental degradation. We could infer this from the sizes of the effects for our country-by-country analysis of the data (see Fig. 3) and from the multilevel logistic regressions (Table 8).

Collectively, our empirical results suggest that in most countries, citizens hold farmers responsible for climate change mitigation and environmental protection if they consider agriculture as a source of climate change and environmental degradation. Although we were not able to test empirically the existence of a direct relationship between perceived cause and treatment responsibility, our findings align with those reported in the existing literature on the attribution of responsibility for environmental and climate protection.

In a study on pesticides, Milford et al. (2022) show that Norwegian consumers attribute responsibility for environmental protection to farmers rather than to themselves. The authors' findings are very consistent with ours on farmers’ perceived responsibility for climate change mitigation and environmental protection. Similarly, Howley et al. (2014) find that the majority of respondents in a survey study in Ireland attributed the responsibility to farmers of using only environmentally friendly farming practices and limiting the use of pesticides and fertilizers. Ricart et al. (2019) examine the results of Eurobarometer surveys since 2008 and also show that European citizens have consistently regarded farmers as responsible for mitigating climate change.

In terms of our second focal explanatory variable, our results suggest that citizens’ political orientation is associated with whether they attribute responsibility for climate and environmental protection to farmers, as left-leaning individuals appear more likely than right-leaning individuals to hold farmers responsible.

This is in line with relevant research that has found a relationship between political ideology and environmental attitudes—be it in single case studies, such as in Sweden (Harring & Sohlberg 2017), or in cross-country studies (Birch 2020; Boer & Aiking 2023). We contribute to this literature by investigating the mediating effect of Ecological and Agrarian parties on this relationship. We show that differences between ideologically left- and right-leaning people in their views on the role of farmers in climate and environmental protection are even more pronounced when such parties are in parliament. This finding is consistent with studies on the impact of party polarization, such as Birch’s (2020), which shows that in the United States, party polarization on environmental policy has amplified public left–right divides on environmental protection. Similarly, J. Kenny (2024) shows for the United Kingdom that politicization of the environment has increased at both the political party and individual levels.

## Conclusion

Policymakers in the EU have increasingly realized that they need to tackle the greenhouse gases originating from agricultural activities (Alons 2017; Bazzan et al. 2023; Schebesta & Candel 2020; Schmidt 2020). Their attempts to adopt corresponding policy measures sparked a wave of farmer protests in late 2023 and early 2024. Against this backdrop, we asked to what degree the public attributes farmers’ responsibility for tackling climate change and environmental degradation in the EU member states and whether the respondents’ perceptions of the causes of these issues and their political ideology can explain to what degree they regard farmers responsible. We further argued that the existence of Agrarian and Ecological parties moderates the hypothesized impact of ideology.

Our findings revealed that perceptions of the causes of climate change and environmental degradation indeed matter for attributing farmers said responsibility. In our view, this finding underpins the importance of differentiating between causal responsibility and treatment responsibility, as suggested by Iyengar (1989). At the same time, we contribute to the pertinent literature, beyond confirming the accuracy of this concept, by showing that the public perceives the two types of responsibility as mutually connected, and in particular by showing that this connection is remarkably robust to differences in the country contexts. Indeed, this covariate was significant in the majority of countries studied, suggesting that individuals have greater odds of attributing farmers’ responsibility if they consider agriculture to be a source of climate change and environmental issues.

Our analyses also hinted at the expected positive relationship between left-leaning individuals and holding farmers responsible for climate and environmental protection, and this relationship was further supported by our multilevel analysis.

On closer inspection of the estimation findings for this covariate, we discovered that the existence of electorally successful Agrarian and Ecological parties matters. Interestingly, we observed a difference in the strength of the effects of ideology dependent on whether such parties were represented in parliament. The differences in the probability of left-leaning vs. right-leaning respondents to attribute responsibility to farmers were more marked in the data for countries in which such parties are represented in parliament. The differences between ideologically left-leaning and right-leaning parties were much more moderate in countries where neither Agrarian nor Ecological parties are represented. This suggests that the existence of these two parties correlates with a higher salience of agriculture and environmental and climate action. Higher salience of these issues may have resulted in the polarization of individuals over the question of how strictly farmers and farming should be regulated in order to fight climate change and environmental degradation.

This finding aligns with those reported, for instance, by M. Kenny and Luca (2021), who have shown for 30 European states that social and political attitudes vary in urban and rural areas.

Despite the insights provided by this study, we are aware that it suffers from limitations which primarily result from the dataset used. The most important limitation concerns the fact that we could not assess directly whether individuals support Agrarian or Ecological parties, but had to rely on their self-placement on a general left–right scale. People voting for Ecological parties tend to be left-leaning, whereas those voting for Agrarian parties tend to be right-leaning (Carter 2013; Mamonova & Franquesa 2020). While support for these two parties maps nicely onto the left–right scale, the measurement is still imperfect, and we invite future research to strive for a more refined measurement of the ideological orientation of individuals. Such a refinement would be particularly welcome for respondents residing in countries with parties that combine ecological and agrarian issues, such as in Latvia. Another evident limitation of this study is its exclusive focus on farmers, which came at the cost of neglecting other actors. In this context, the study by Milford et al. (2022) is worth highlighting because the authors distinguish between the responsibility of farmers, consumers, and public authorities. An outcome variable with additional categories would have made the analysis more wholesome and intriguing. While we acknowledge the omissions of the present analysis, we are confident that it offers an apt point of departure for future studies that would improve our study’s theoretical perspective and empirical analysis.

Overall, our findings of a relationship between perceived cause and treatment responsibility for farmers to take environmental and climate action and a moderating effect of Agrarian and Ecological parties on the relationship between political ideology and environmental attitudes provide valuable insights into how EU citizens perceive and respond to the EU’s agenda to make agriculture more sustainable. This, in turn, can help decision-makers better understand resistance to the EGD and help them overcome barriers to a green transformation of agriculture.

## Data Availability

The data is available via Heidelberg University’s institutional research data repository heiDATA: https://doi.org/10.11588/data/3M7HXT
